# Digital leadership: Norwegian healthcare managers’ attitudes towards using digital tools

**DOI:** 10.1177/20552076241277036

**Published:** 2024-09-05

**Authors:** Jannike Dyb Oksavik, Erlend Vik, Ralf Kirchhoff

**Affiliations:** 1 Department of Health Sciences, Aalesund, 8018 Norwegian University of Science and Technology, Aalesund, Norway; 2 Faculty of Business Administration and Social Sciences, 5562 Molde University College, Molde, Norway

**Keywords:** Healthcare management, digital leadership, digital tools, primary health care, hospital, organization and administration

## Abstract

**Background:**

Health services are undergoing digitalization and applying new digital tools. These changes may provide healthcare managers with opportunities to exercise digital leadership. However, managers’ attitudes may influence the extent to which they demonstrate digital leadership. This study explores the attitudes of Norwegian healthcare managers towards: (1) digital tools and change and (2) to what extent digital tools are applicable to various tasks of managers.

**Methods:**

Cross-sectional study including 154 managers in hospitals and municipal health services in a Norwegian county. The questionnaire was about management and digital tools, and the data was analyzed by descriptive statistics, correlations, and content analysis.

**Results:**

The healthcare managers perceived that digital tools facilitated a positive change in organizational work processes aligned with values and goals. Digital tools supported administrative tasks such as gaining control over responsibilities. However, 76 managers stated that certain tasks, including interactions with employees (e.g. performance appraisals and sick leave follow-up) and the building of an organizational culture, should not be performed using digital tools or using them only to a limited extent; for these tasks, they preferred in-person meetings.

**Discussion:**

Norwegian healthcare managers’ attitudes toward digital tools are generally positive, but there are areas where they find the tools less suitable.

**Conclusions:**

The results provide new insights into healthcare by indicating that many managers may have positive attitudes toward digital tools. However, digital leadership may not be applicable equally in all areas of healthcare managers’ work. This raises the question of whether digital leadership can or should be exercised uniformly in every area of health services.

## Introduction

Health services are constantly changing through digitalization and adoption of digital tools.^1–4^ The technologies that many Western countries in the early 2000s referred to as “advanced information technology” are now standard applications in healthcare.^5,6^ Digital tools are crucial in meeting challenges like the COVID-19 pandemic, the increasing number of patients, and the high cost of health services.^4,7–9^ Today, multiple digital tools, such as quality management systems, electronic health records, and communication software like Microsoft Teams, are used in the work of healthcare managers.^4,10^ Moreover, digital tools need to be put to productive use, for which healthcare managers are particularly responsible.^8^ Hence, managers’ attitudes toward digital tools and assessments of their usefulness are essential to examine for extending knowledge of digital leadership in practice.

There are knowledge gaps in the literature regarding leadership in digital contexts; there have been few studies of digital leadership in healthcare.^11^ In practice, healthcare managers may need more preparation to lead, as their knowledge of digitalization and implementation of digital tools may be greater than that of their subordinates.^1^ In line with other authors,^1–3^ we understand digital leadership as leading in a digital context. Digital leadership encompasses several overlapping concepts, including virtual leadership, which is computer-mediated communication, and e-leadership, which is a social influence process in digital transformation.^2,12^ Research on digital leadership can be divided into four areas: (1) leading digital transformation, (2) task achievement using digital tools, (3) an interpersonal orientation of leadership, and (4) personal aspects of the manager that may influence digital leadership.^2^ These areas will be briefly outlined in the following section, delimited to how they relate to leadership and digital tools.

### Digital leadership and digital tools

Firstly, digital leadership considers the process of digital transformation.^3,10^ Digital transformation goes far beyond technical change; it also requires an adaptive shift in organizational cultures, including in the attitudes of health professionals and managers.^8,13^ Healthcare managers are key actors in digital transformation and organizational change; however, few studies have examined their attitudes toward digital tools. Attitudes are formed by beliefs, values, and experiences and can change over time.^14^ Scholars have debated to what extent attitudes influence behavior, but attitudes toward a phenomenon more strongly influence behavior if combined with direct experiences and behavior-relevant information.^15^ Attitudes toward adopting technologies are dependent on various aspects. Studies have found that the perceived usefulness of tools is important. For example, attitudes toward using digital tools can be influenced by whether the tools improve jobs or outcomes such as service delivery. Resistance to change may also occur, for example, if actors perceive the change is unaligned with personal and organizational values and goals. Attitudes to discrete tools can be shaped by overall attitudes to digital transformation in society.^13,16^

Secondly, digital leadership involves using technology in task achievement.^2,3^ Administrative tasks designed to produce predictability and order in the organization are essential to leadership.^17^ Digital tools, such as enterprise systems or software, are intended to give healthcare managers an overview and control of various tasks and responsibilities, such as deviations in service delivery, finances, and employee performance.^9^ Moreover, new electronic health records may provide managers with opportunities for data analytics related to health service delivery.^10^

Thirdly, digital leadership has an interpersonal dimension, such as when managers interact successfully with others, communicate digitally, and foster trust-based relationships.^2^ Digital leadership is also linked to distance or remote digital work conducted through, for example, video conferencing, digital meetings, and email correspondence.^18^ However, building and maintaining trust can be difficult for managers using digital tools.^2,11^

Fourthly, a manager's background and personal characteristics may influence digital leadership. Research has been conducted on the individual traits of managers, such as adaptability,^2^ and manager roles, such as advocating for change,^1^ that positively affect digital leadership. Simultaneously, there is scant knowledge about how other management conditions are associated with managers’ attitudes to digital tools. For example, do associations exist between managers’ attitudes and their work experience, working context, age, or level of education? A manager's span of control, that is, the number of subordinates one is responsible for, could also be associated with attitudes toward digital tools because it may be challenging to follow up and communicate with a large number of employees^19^; therefore, there could be differences in levels of digital leadership related to the number of subordinates a manager oversees. Finally, managers’ characteristics may also influence their attitudes to the abovementioned areas of digital leadership.

The aim is to explore the attitudes of Norwegian healthcare managers toward digital tools within the abovementioned areas. Norway is an interesting context because it has adopted several digitalization strategies, such as new standards for electronic health records and structured information sharing (Norwegian Ministry of Health and Care Services^20^ pp. 29–32,^21^). The attitudes of healthcare managers are essential in motivating employees to use and adopt digital tools and in successfully adapting them in their organizations.

Based on the four areas of digital leadership, the following research questions will be explored:

1. What attitudes do Norwegian healthcare managers have toward change because of digital tools?
2. To what extent do healthcare managers find digital tools applicable to various tasks?

## Methods

The cross-sectional study was conducted in Western Norway, a county with approximately 270,000 inhabitants. Norway has a tax-based national health system with semidecentralized governance. The state governs hospitals, while the municipalities are responsible for primary healthcare services such as nursing homes and home care services.^22^ Therefore, we included managers in both hospitals and municipalities. At the time of the study, this county had started implementing digital tools such as remote patient monitoring sensor technology. It was in the first implementation phase of a new electronic health record to increase health service integration and give healthcare managers new functionalities to analyze data from the health services.

We collected the data for the survey in September 2022 using two different sampling strategies in hospitals and municipalities, respectively. In the four public hospitals in the county, all 200 managers at different levels were invited to participate in the survey by email. In the 26 municipalities, we found the email addresses of approximately 300 managers. However, contact information for many managers was unavailable on municipal web pages. Hence, the municipal sample was convenient as the total number of managers working in the region is unknown. In sum, we invited 500 healthcare managers to complete the survey and sent a reminder by email after one week. The respondents could not be identified because the software/webpage for data collection made tracking IP addresses impossible. We used a web-based survey tool called Nettskjema, developed by the University of Oslo. It allows the creation, storage, and management of surveys and data collection.

### The questionnaire

JDO and RK developed the questionnaire for this study and it was not validated. Our work was explorative because the concept of digital leadership by using digital tools in managerial work was not operationalized when we planned the study. We have experience in developing questionnaires, and collaborated with colleagues at a master's program in health management and two clinical managers (see Acknowledgments). The digital leadership literature inspired the questions we developed about managers’ attitudes toward digital tools. The survey measures cover the four different areas of digital leadership: digital transformation, task achievement, interactions with others, and manager characteristics.

The survey items related to the digital leadership area “digital transformation” were “Implementing digital tools improves work processes”; “Changes resulting from the use of digital tools is overall positive for my unit”; “Changes resulting from the use of digital tools contribute to fulfilling the goals of my organization”; and “Changes resulting from the use of digital tools is consistent with my professional values.”

For the second area of digital leadership, “using digital tools for task achievement,” we used six items (e.g., “ Digital tools help me stay abreast of the area I am responsible for.”) to measure the perceived usefulness of digital tools for management tasks. These items covered an overview of finances, employee performance and deviations, digital tools for analysis, and working effectively. These items are displayed in Table 2 in the results section. The items could be answered on a 7-point Likert scale ranging from 1 = *strongly disagree* to 7 = *strongly agree*, with “not applicable” also available as an option. To explore whether digital tools were perceived as less suitable or unsuitable for any areas, we asked an open-ended question: “Are there any managerial tasks that you think should be carried out without digital tools or using digital tools to a lesser extent?”

For the digital leadership area “aspects of the manager,” we included background variables: *work context* (primary healthcare or hospitals), *formal education in management* (yes/no), *work experience as a manager in years* (1–3, 4–10, 11–20, or >20 years), *age* (<35, 35–44, 45–54, or ≥55 years), *educational level* (university/bachelor's degree, university/master's degree, and other) and the *span of control* meaning the number of subordinates reporting to the manager (1–500 employees).

### Analysis

The data was analyzed using IBM SPSS Statistics software version 28.0.1.0 (142). We checked the data for a normal distribution. Then, we used frequency analysis, two-tailed *t*-tests to compare groups, and correlation analyses. One-sided correlation (Spearman's rho) was used for nominal background and attitude variables. Pearson's correlation was used for continuous variables.

The four items related to “digital transformation” had a covariance (Cronbach's alpha) of .885 in our survey. These items were added to a scale labeled “attitudes toward change resulting from the use of digital tools.” For each respondent, we calculated a total score on this scale. Then, we performed a correlation analysis with the demographic variables, using Spearman's rho for nominal and ordinal variables and Pearson's correlation for continuous variables. Correlations between 0.2 and 0.39 were considered “weak.”^23^ In the correlation analyses, responses of “not applicable” were excluded.

We did a content analysis^24^ of the answers to the open-ended question, “Are there any managerial tasks that you think should be carried out without digital tools or using digital tools to a lesser extent?.” The analysis was done in WordStat version 9.^25^ We coded each answer into a managerial task and subsequently categorized them into groups of tasks (e.g. responses like "performance appraisals require physical presence” were placed in the category “performance appraisals”). At the final stage, we counted occurrences of each task. 

## Results

The questionnaire was returned by 154 managers for a response rate of 35%. Of these responding managers, 85 worked in primary health care and 70 in hospitals. The typical respondent was a female middle manager with a span of control of 76–79 employees. Table 1 describes the study participants.

**Table 1.** Study participants.

Manager characteristics	Frequency/percentage distribution
**Age**	
<35	13 (8.4%)
35–44	44 (28%)
45–54	34 (22%)
≥55	62 (40%)
Missing	1 (0.6%)
**Gender**	
Female	120 (78%)
Male	33 (21%)
Other	1 (0.6%)
**Manager experience**	
1–3 yr	45 (29%)
4–10 yr	49 (31%)
11–20 yr	31 (20%)
>20 yr	27 (17%)
Missing	2 (1.2%)
**Education**	
Nurse	78 (50%)
Medical doctor	10 (6%)
Other	66 (42%)
**Management education**	
Yes	84 (54%)
No	55 (35%)
**Taking a management course now**	14 (9%)
Missing	1 (0.6%)
**Span of control**	
3–500 employees	Mean 77

### Digital tools and change

The managers’ attitudes were positive, overall, regarding whether digital tools could facilitate change aligned with organizational goals and personal values. They agreed in four statements: (1) Implementing digital tools improved work processes. Changes resulting from the use of digital tools were (2) positive for their unit, (3) the changes contributed to fulfill the goals of their organization, and (4) were consistent with their professional values.

These four statements were added into a scale and the mean added value was 5.5, in which value 1 indicated “*strongly disagree*,” and 7 was “*strongly agree*.” The responses in this scale, labeled “attitudes toward change resulting from the use of digital tools,” correlated with some of the individual characteristics of the managers. Individual characteristics that were weakly associated with positive attitudes toward change resulting from the use of digital tools were having worked more years as a manager (Spearman's rho *r* = .177, *p* = .028, *n* = 153), being older (Spearman's rho *r* = .186, *p* = .021, *n* = 154), and having a higher span of control (Pearson's correlation *r* = .188, *p* = .019, *n* = 154). Managers at higher levels of health services tended to be more optimistic about change resulting from using digital tools.

T-tests (two-tailed) showed no differences in attitudes between managers across genders or levels of education (bachelor's degree vs five or more years of education), nor did having an education in management influence attitude scores. Moreover, we found no difference between managers who worked in municipalities and hospitals.

### Digital tools for administrative tasks

The perceived usefulness of digital tools for administrative tasks was high, towards the top of the scale from 1 to 7, as shown in Table 2.

**Table 2.** Attitudes toward digital tools in managerial work.

	mean	SD
Digital tools help me stay abreast of the area I am responsible for (*n* = 154)	6.49	.818
Digital tools help give me an overview of finances in the unit I am responsible for (*n* = 147)	6.31	1.208
Digital tools help me work more effectively (*n* = 154)	6.24	1.054
Digital tools help me have an overview of the performance of my employees (*n* = 150)	5.10	1.678
I use digital tools for analysis purposes (*n* = 150)	5.07	1.723
I benefit from digital tools in following up on deviations (*n* = 153)	6.39	1.027

### Digital tools for interactions

The managers tended to neither agree nor disagree with the statement, “I communicate more digitally than physically with my employees.” The mean value was 3.57 on a scale of 1 to 7, and the SD was 1.713. This statement positively correlated with the managers’ span of control (*r* = 3.98, *p* = .001, *n* = 154).

The managers reported that some tasks should be carried out without digital tools or using them to a lesser extent. Seventy-six managers stated that digital tools should not be used to assist with certain work tasks. The responses to the open-ended question, “Are there any managerial tasks that you think should be carried out without digital tools or using digital tools to a lesser extent?” were assigned to categories. Table 3 shows the number of occurrences per category.

**Table 3.** Tasks to be carried out without digital tools/using digital tools to a lesser extent.

Task	Frequency
Collaboration	5
Job interviews	1
Meetings	14
Following up next of kin/patients	2
Performance appraisals	26
Sick leave follow-up	6

Some managers described the limitations of digital tools for their interactions with employees:

Digital tools can mainly be used for administration, but building a good culture through digital platforms is more challenging. The digital leader is challenged to exercise leadership through presence while using digital platforms simultaneously, which is essential for good leadership.

Digital tools cannot replace the manager's presence among employees. It is a necessary supplement that simplifies many management tasks.

Conversations with my employees must be in person. Employees must experience being seen.

Digital tools should not replace a good conversation. In addition, our employees will measure their managers by what they do—not by what is filled out on a form. Remember that we must solve specific tasks—then the fact that a form ‘demands to be filled in’ without anyone asking for this can work against its purpose.

Other managers had both physical and digital meetings with their employees:

It is important that we continue to have physical meetings with employees—a combination of digital and physical should also be maintained in the future.

An excellent tool when collaboration with employees is not possible. When we have a ward meeting, the employees who cannot attend physically can participate digitally.

Others mentioned that this was important in a context where many worked shifts and that digital tools increased the manager's opportunity to reach out to all employees.

Digital tools are suitable for administrative tasks, and leadership should occur directly with human contact.

Three managers answered that all tasks could be carried out using digital tools.

## Discussion

To a high extent, healthcare managers in the Norwegian region agreed that digital tools could facilitate change in their organizations. We found weak correlations between these positive attitudes and the age of managers (*r* = .186) and their span of control (*r* = 1.88) but not with other individual or contextual variables. Digital tools supported administrative tasks such as gaining control over responsibilities. Some managers considered digital tools less suitable for interpersonal tasks like performance appraisals, meetings, and follow-up of employees during sick leave. However, the managers mainly highlighted the advantages of digital follow-up of employees.

These findings indicate that the conceptual idea of digital leadership—leading in a digital context^1–3^—appeared to varying extents in managers’ attitudes. The attitudes of healthcare managers varied with the different aspects of leadership and digital tools. Two aspects will be discussed: How/if digital leadership was visible in managers’ attitudes toward change; and to what varying degrees in different work tasks.

### Attitudes to change

We interpret the attitudes to change because of digital tools being overall positive among the managers. Few studies of healthcare managers’ attitudes toward digital tools exist. The positive attitudes of managers align with previous studies on healthcare staff.^5,6^ Of course, managers may feel obliged to project positive attitudes toward digital tools because they often have formal responsibilities for digital transformation within their organizations.^13^ However, two aspects making digital leadership easier could be considered when interpreting why the managers’ attitudes were positive: changes in the healthcare context and managers’ span of control.

Firstly, the changes in the healthcare context that could have influenced our results are the COVID-19 pandemic and previous and ongoing digitalization processes in Norwegian healthcare. The emergency of the COVID-19 pandemic facilitated digital leadership by increasing the use of digital tools.^4,7^ For example, according to a report from Microsoft, the use of Microsoft Teams increased by 252% during the pandemic (Microsoft,^26^ p. 2). A disruptive change has occurred in the increased use of digital communication and digital tools.^4^ Also, the digital work of Norwegian managers started to change before the pandemic. The government has launched digitalization reforms for the Norwegian public sector.^21^ In health services, this entails digitalization of care coordination, increased use of digital tools, and a belief that technologies can improve the efficiency of health services and save costs.^20^ The region where this survey was conducted had started implementing new digital tools such as remote patient monitoring, sensor technology, and electronic health records with new functionalities for managers.

Secondly, the span of control among managers in this study averaged almost 80, and we found an association between the span of control and the degree of reliance on digital communication with employees. Digital tools can make the follow-up of many employees easier, for example in digital meetings. Managers with a high span of control have many tasks, especially involving administrative work and follow-up of employees. A high span of control can create communication and coordination challenges.^19^ The location of our study was a county with rural areas where geographical distances can pose challenges, and technology can reduce travel time for managers and employees. Still, we found only weak correlations between the span of control and attitudes toward change resulting from using digital tools. This may indicate that managers find digital tools useful regardless of their span of control.

### Digital leadership in work tasks

Managers perceived that digital tools helped them complete their administrative tasks, such as gaining control, achieving an overview, and improving efficiency in their work. This may not be surprising, considering research showing that new technologies are helping managers carry out more advanced data analytics and increasing the efficiency of health service delivery.^9^

However, considering that the digital leadership role as it is described in the literature involves both managing administrative tasks and leading people digitally,^3,17^ there may be a gap between the literature and healthcare practices in that many managers found digital tools unsupportive to the relational dimension of their leadership role. The conceptual idea of pure digital leadership, which is understood as using digital tools to lead and manage people, processes, and organizations,^1–3^ emerged differently in practice, as several managers in both primary health care and hospitals regarded meeting in person as essential. A possible explanation for the negotiation of the digital, could be that managers have a sense of care and responsibility for their employees. Our respondents generally preferred doing administrative tasks digitally but were more ambivalent about using digital tools to accomplish tasks involving direct leadership of employees. Within the healthcare context, employees handle complex priorities, e.g. limited resources, and even face life-and-death situations. Some studies point to “the dark side” of digital work environments related to psychological strain, stress, isolation, and human relations.^7,27^ It could be more challenging to prioritize and lead people in healthcare than, for example in the industry.

Conversely, a literature review taking an employee perspective suggests a positive association between virtual leadership and well-being and job satisfaction and a negative association with psychological strain, stress, and perceptions of isolation in digitally collaborating employees.^27^ Three of our respondents envisioned that digital leadership could be carried out in the pure form. We assume multiple ways of combining digital and in-person leadership may occur across contexts.

### Study limitations and strengths

The results are not generalizable to healthcare managers because of the small sample size, the response rate of 35%, and the fact that the sample consisted mainly of mid- and front-line managers. We used no validated survey instrument, which is a limitation in measuring the concept of digital leadership; the construct validity is limited. However, digital leadership in healthcare through digital tools was to a less extent operationalized in the literature, and a strength of the study is that it could be interpreted as being explorative in this area.

### Suggestions for further research

Future studies could use a representative sample and a longitudinal perspective to extend existing knowledge of digital leadership development. It would also be interesting to explore how digital leadership can be exercised in different areas of healthcare managers’ work and what kinds of digital interactions make digital leadership successful.

## Conclusions

The attitudes of healthcare managers were positive toward the organizational changes that digital tools could bring about. The perceived usefulness of digital tools was high for the achievement of administrative tasks and lower for the interpersonal dimension of digital leadership. The attitudes of managers in a healthcare setting differ from the digital leadership literature, depicting leadership as a phenomenon and process that can occur digitally. The concept of digital leadership, understood as leading and managing in a digital context, may not be appreciated in all areas of healthcare managers’ work, which raises the question of whether healthcare managers will carry it out. Managers in a healthcare context may take a situational approach to digital tools and a hybrid leadership approach that combines digital and face-to-face communication.

## The questionnaire—digital leadership

Introduction text to respondents:

We live in a time where high expectations are placed on you who are managers in the health and care services. In this survey, we would like feedback on how you as a manager experience various conditions related to digitization and change in the health and care services. More knowledge is needed about managers’ experiences in this area.

Think about the digital tools that are important to you as a manager. Rate how much you agree or disagree with the statements below. Please answer based on your role as manager and the tasks you must solve. If any of the statements are not applicable to your work as a manager, tick “Not applicable.”

### Digital tools and change

Implementing digital tools improves work processes.

Changes resulting from the use of digital tools is overall positive for my unit.

Changes resulting from the use of digital tools contribute to fulfilling the goals of my organization.

Changes resulting from the use of digital tools is consistent with my professional values.

These items could be answered on a 7-point Likert scale ranging from 1 = *strongly disagree* to 7 = *strongly agree*, with “not applicable” also available as an option.

### Digital tools and administrative tasks

1. Digital tools help me stay abreast of the area I am responsible for.
2. Digital tools help give me an overview of finances in the unit I am responsible for.
3. Digital tools help me work more effectively.
4. Digital tools help me have an overview of the performance of my employees.
5. I use digital tools for analysis purposes.
6. I benefit from digital tools in the following up on deviations.

These items could be answered on a 7-point Likert scale ranging from 1 = *strongly disagree* to 7 = *strongly agree*, with “not applicable” also available as an option.

### Interactions with others

**“**I communicate more digitally than physically with my employees.” This item could be answered on a 7-point Likert scale ranging from 1 = *strongly disagree* to 7 = *strongly agree*, with “not applicable” also available as an option.

“Are there any managerial tasks that you think should be carried out without digital tools or using digital tools to a lesser extent?”

### Background variables

work context (primary healthcare or hospitals)

formal education in management (yes/no)

work experience as a manager in years (1–3, 4–10, 11–20, or >20 years)

age (<35, 35–44, 45–54, or ≥55 years)

educational level (university/bachelor's degree, university/master's degree, and other)

span of control (1–500 employees).
